# The Unexpected Detection of Esophageal Varices Caused by Liver Cirrhosis in a 47-Year-Old Man Treated with a Growth Hormone in Childhood

**DOI:** 10.3390/diseases12100251

**Published:** 2024-10-12

**Authors:** Osamu Arisaka, Satomi Koyama, George Imataka, Junko Naganuma, Takahiro Arisaka, Sei Akatsuka

**Affiliations:** 1 Department of Pediatrics, Dokkyo Medical University, Tochigi 321-0293, Japan; s-koyama@dokkyomed.ac.jp (S.K.); geo@dokkyomed.ac.jp (G.I.); i-junko@dokkyomed.ac.jp (J.N.); 2 Department of Gastroenterology, Dokkyo Medical University, Tochigi 321-0293, Japan; aritaka@dokkyomed.ac.jp; 3 Department of Pediatrics, Koto Hospital, Tokyo 136-0072, Japan

**Keywords:** childhood, growth hormone deficiency (GHD), metabolic dysfunction-associated steatotic liver disease (MASLD), metabolic dysfunction-associated steatohepatitis (MASH), esophagus varices

## Abstract

*Background:* We report a rare case highlighting the progression of liver disease in a male patient with idiopathic childhood-onset growth hormone (GH) deficiency. *Case presentation:* The patient was diagnosed with hypopituitarism at six years old and was treated with thyroxine therapy and GH for his short stature, with testosterone added at the age of 15. GH therapy was discontinued when the patient was 18 years old, but thyroid and testosterone treatments continued. The patient had been taking medication for hyperlipidemia until the age of 30 and was noted to have impaired glucose tolerance at the age of 40, but HbA1c levels remained normal. At the age of 47, esophageal varices were incidentally discovered via endoscopy, revealing liver cirrhosis. Laboratory tests showed liver dysfunction and abnormal lipid levels, and hepatitis viral markers were absent. The patient had no history of drinking alcohol or smoking, and no family history of diabetes. *Results:* Ultimately, this case demonstrates that metabolic dysfunction-associated steatotic liver disease (MASLD/metabolic dysfunction-associated steatohepatitis (MASH)) is under-recognized in GH deficiency cases and can progress to liver cirrhosis. *Conclusions:* Therefore, careful evaluation of MASLD/MASH in childhood-onset GH deficiency is necessary, and GH replacement therapy should continue into adulthood, if possible.

## 1. Introduction

Metabolic dysfunction-associated steatotic liver disease (MASLD) is defined by the presence of steatosis in over 5% of hepatocytes associated with metabolic risk factors such as obesity and type 2 diabetes, and with the absence of excessive alcohol consumption and/or other chronic liver diseases. With the increasing prevalence of obesity, MASLD has become a common cause of chronic liver disease [1]. MASLD is a disease on a spectrum spanning from simple benign steatosis to metabolic dysfunction-associated steatohepatitis (MASH) with fibrosis and scarring that can eventually lead to cirrhosis [2,3]. 

MASLD is highly prevalent and associated with significant adverse outcomes through both liver-specific morbidity and mortality and, perhaps more importantly, adverse cardiovascular and metabolic outcomes. It is closely associated with type 2 diabetes and obesity, and both conditions may progress to more advanced stages. The mechanisms that govern hepatic lipid accumulation and the predisposition to inflammation and fibrosis are still not fully understood but reflect a complex interplay between metabolic target tissues, including adipose and skeletal muscle, and immune and inflammatory cells [2,3].

While the association between MASLD and obesity has been well documented, endocrine disorders such as growth hormone (GH) deficiency, hypothyroidism, hypogonadism, and polycystic ovarian syndrome are known in clinical practice to be associated with MASLD [4,5].

In this study, we report a patient with idiopathic childhood-onset GH deficiency who received GH to treat his short stature during childhood. The clinical course progression of liver lesions (from MASLD to MASH) was unnoticed until the incidental discovery of esophageal varices due to liver cirrhosis. This study aims to report the clinical course of a patient and review the current knowledge on the pathophysiologic mechanisms of GH in MASLD development.

## 2. Case Report

The patient was born via breech delivery and with asphyxia at 40 weeks of gestation at a weight and height of 2320 g and 45 cm, respectively. He was delivered as a small-for-gestational-age (SGA) infant (weight below the 10th percentile for his gestational age). At six years of age, he was referred for treatment because of his short stature. At that time, he was 95.0 cm in height (−3.5 SD on the growth chart for Japanese boys) and 16.0 kg in weight (−1.7 SD). He had normal body proportions. Routine laboratory analysis ruled out hematologic, liver, and renal diseases. An endocrinological examination showed extremely low GH responses (<5 ng/mL) to provocation tests with insulin and arginine. Serum somatomedin-C (insulin-like growth factor-I, IGF-I) could not be measured at the time as no measurement method had been established.

The patient was diagnosed with hypopituitarism, presenting with deficiencies in growth, thyroid-stimulating, luteinizing, and follicle-stimulating hormones. Pituitary-extracted human GH (*phGH*) injections and oral thyroxine were subsequently started.

Intramuscular testosterone injections were started at the age of 15 to promote secondary sex characteristics (the patient’s final penile development stage was Tanner 3). GH therapy was continued only until the age of 18 (in the year 1985), but thyroid hormone administration and regular testosterone injections were continued thereafter. Regular blood tests during GH therapy revealed no abnormalities in liver function or serum lipid levels. His neurological and psychomotor developments were normal.

After the patient became an adult, he had to move for work and received follow-up treatments at another hospital. He lived a normal daily life without any notable problems. From the age of 30, he received drug treatment for hypertriglyceridemia. Impaired glucose tolerance was noted at 40 years of age, but glycated hemoglobin (HbA1c) levels remained at 4.9–5.6% (normal, <5.2%) with sufficient exercise and diet therapies.

**Figure 1:**
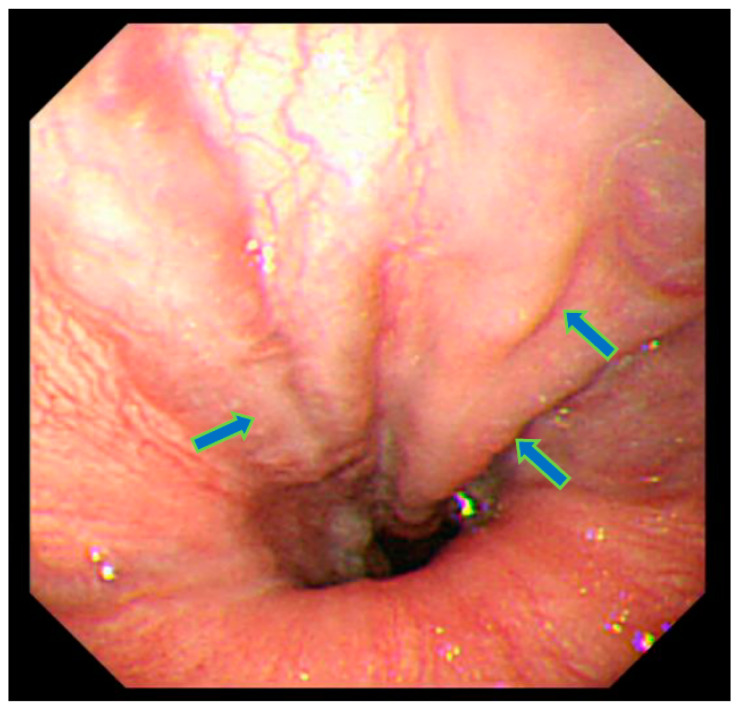
Upper gastrointestinal endoscopy showing esophageal varices in the lower esophagus (arrows). Varicose veins have the same color as normal esophageal mucosa. Bleeding or redness on the surface was not recognized. Judged to be grade 1.

At age 47, the patient experienced stomach discomfort and underwent upper gastrointestinal endoscopy, which incidentally revealed esophageal varices (Figure 1). There was no relationship between the stomach discomfort and the esophageal varices. His height was 155.2 cm (−2.5 SD) and his weight 72.2 kg (body mass index: 29.9 kg/m^2^). The patient was not married and worked as a computer engineer. He had no family history of diabetes mellitus and no history of a drinking or smoking habit. In a physical examination, there were no cutaneous changes indicating extrahepatic manifestations of palmar erythema or spider angioma. Moreover, dilated superficial abdominal vein and hepatosple nomegaly were not observed at that time.

The laboratory findings showed the following measurements: 4940/μL of WBC (neutro: 62.0%; lymph: 26.9%; mono: 7.7%; eosino: 2.8%; and baso: 0.6%), 16.8 g/dL of Hb, platelets of 12.7 × 10^4^/μL, prothrombin activity of 65% (normal levels: 80–120%), 47 IU/L of AST (normal levels: 10–35 IU/L), 25 IU/L of ALT (5–35 IU/L), 151 IU/L of γ-GTP (10–50 U/L), 304 IU/L of LDH (124–220 IU/L), 231 IU/L of ChE (210–550 IU/L), 1.3 mg/dL of total bilirubin, 0.6 mg/dL of direct bilirubin, 3.3 g/dL of albumin (3.8–5.3 g/dL), 67 μg/dL of ammonia (12–66 μg/dL), 14 μmol/L of total bile acids (<10 μmol/L), 140 mg/dL of LDL-cholesterol (65–139 mg/dL), 26 mg/dL of HDL-cholesterol (>40 mg/dL), and 126 mg/dL of triglyceride (30~140 mg/dL). An endocrinological examination revealed 4 ng/dL of IGF-I (90–250 ng/dL), 1.09 ng/dL of free thyroxine at 5.4% HbA1c (0.7–1.48 ng/dL), and 236 ng/dL of testosterone (264–916 ng/dL). No hepatitis virus markers were detected, including hepatitis B and C. The antinuclear antibody titer ×40 (<1:80) test for liver fibrosis yielded the following measurements: 125 ng/mL of hyaluronic acid (normal levels < 50), an APRI of 1.06 (cut-off value of 0.7), and a Fib-4 index of 3.48 (cut-off value of 2.04).

**Figure 2:**
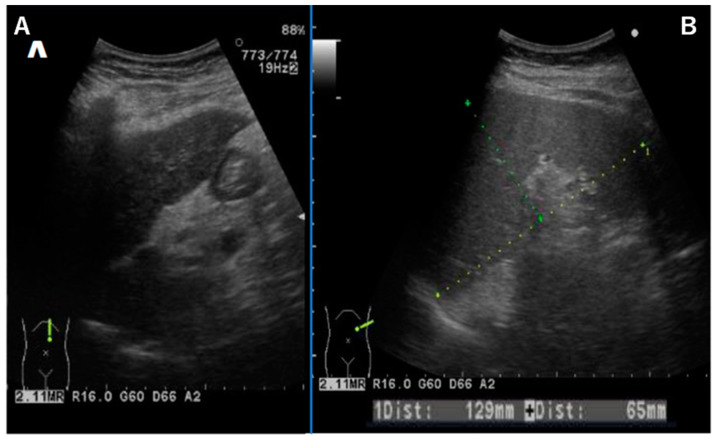
Abdominal ultrasound showing a liver contour with an irregular appearance (**A**). The spleen was enlarged, and ascites were absent (**B**).

Abdominal ultrasound showed a liver contour with an irregular appearance, consistent with the findings of liver cirrhosis (Figure 2A). The spleen was enlarged, and ascites were absent (Figure 2B). Esophageal varices were thought to be due to portal hypertension. A liver biopsy was not performed because of its associated invasive risks.

## 3. Discussion

In this case, Wilson’s disease and noncirrhotic portal hypertension (NCPH), which can cause liver cirrhosis from childhood, were ruled out based on the current medical history and clinical test data.

GH and IGF-1 are crucial for linear growth during childhood and for maintaining significant metabolic functions throughout adulthood [6]. In this case, hypopituitarism, primarily characterized by GH deficiency, was attributed to pituitary stalk interruption due to a birth injury, although this was not confirmed via brain magnetic resonance imaging [7]. In instances of pituitary stalk interruption, T1-weighted MR imaging can reveal the absence of a pituitary stalk and the presence of an ectopic lobe [8].

The patient commenced GH replacement therapy at age six. Despite this, his final height, measured at −2.5 SD as an adult male, was suboptimal, primarily due to the limited availability of recombinant human growth hormone (hGH) before 1988 [9].

GH and IGF-I are vital for growth in childhood and have important metabolic roles in adults. Adult GH deficiency (AGHD) is associated with increased visceral fat, dyslipidemia, premature atherosclerosis, reduced quality of life, and elevated mortality [10]. Cardiovascular disease, cerebrovascular disease, and malignancy are significant contributors to premature mortality in AGHD patients [10,11].

Recent case studies and clinical research indicate a correlation between AGHD and a heightened prevalence of MASLD, with progression to MASH or cirrhosis [5,12,13]. MASH diagnosis relies on liver biopsy histology, which shows steatosis, inflammatory cell infiltration, hepatocyte ballooning, and fibrosis, potentially advancing to cirrhosis and hepatocellular carcinoma, resulting in a poor prognosis [14,15]. Risk factors for MASLD/MASH include obesity, metabolic syndrome, type 2 diabetes, dyslipidemia, and particularly insulin resistance [12,13].

### 3.1. GH and IGF-1

**Figure 3:**
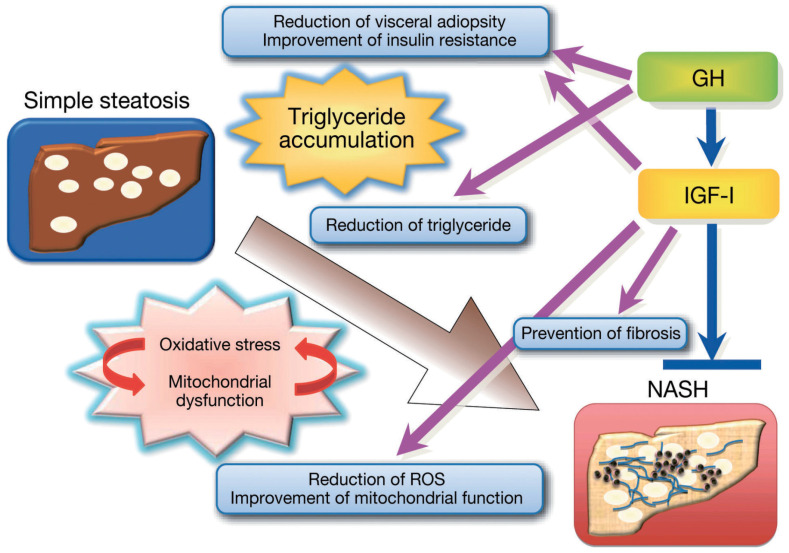
GH and IGF-I actions in the liver. GH improves triglyceride accumulation in hepatocytes, while IGF-I reduces oxidative stress, enhances mitochondrial function, and may prevent fibrosis progression. The figure is adapted from reference [12] (NASH has been renamed as MASH in 2023).

The liver is a significant target organ for GH. Initially, the somatomedin hypothesis suggested that the liver was merely an organ secreting IGF-I [16]. Recent evidence highlights the crucial roles of both GH and IGF-I in the liver, particularly in adults. GH serves as a major metabolic hormone, optimizing body composition and regulating energy and substrate metabolism. It enhances fat metabolism by promoting lipolysis and fatty acid oxidation, indirectly activating hormone-sensitive lipase via β-adrenergic stimulation. GH also boosts low-density lipoprotein (LDL) clearance by increasing hepatic LDL receptor expression [17]. GH affects glucose metabolism directly and by counteracting insulin action and suppressing glucose oxidation and utilization while increasing hepatic glucose production [18]. In protein metabolism, GH reduces urea synthesis, blood urea concentration, and urinary urea excretion while lowering protein oxidation and stimulating protein synthesis; its effects are mainly mediated via IGF-I, though direct GH actions are also recommended [19]. Conversely, IGF-I enhances glucose sensitivity through direct and indirect mechanisms, including the feedback inhibition of GH secretion [20,21,22]. IGF-I strongly promotes protein synthesis and inhibits protein breakdown [23]. Generally, GH and IGF-I work synergistically across various tissues, except for IGF-I’s insulin-like actions (Figure 3).

### 3.2. GH Action in the Liver

GH locally generates IGF-I for autocrine and paracrine effects [24], but circulating IGF-I predominantly originates from hepatocytes [25,26]. Liver-specific GH receptor deletion (GHRLD) in mice led to significantly reduced serum IGF-I levels, with GHRLD mice displaying normal linear growth but reduced bone density like liver-specific IGF-I deficient mice [25]. Notably, GHRLD mice exhibited insulin resistance, glucose intolerance, elevated free fatty acids, decreased triglyceride efflux, and severe steatosis, underscoring the importance of GH signaling in the liver. In humans, GH receptor loss-of-function mutations (e.g., Laron syndrome) also manifest as MASLD, with chronic IGF-I replacement not affecting MASLD status, suggesting GH’s direct role in preventing steatosis in hepatocytes [27] (Figure 3).

### 3.3. IGF-I Action in the Liver

Patients with chronic liver disease and malnutrition show reduced free IGF-I levels despite normal or elevated GH secretion, given that the liver is the primary source of serum IGF-I, as evidenced by GHRLD mice [28]. However, IGF-I does not directly impact hepatocyte function as hepatocytes have few IGF-I receptors under normal conditions [29]. The literature indicates that IGF-IR plays a crucial role in the liver [30]. Nishizawa et al. [31] demonstrated that GH-deficient dwarf rats exhibit MASH, which is reversed via IGF-I and GH administration. IGF-I’s effects on the liver may involve mechanisms, such as improved insulin sensitivity, as IGF-I deletion from the liver results in insulin resistance [32], suggesting that increased levels of circulating IGF-I can alleviate MASH partly by enhancing insulin sensitivity. Additionally, IGF-I’s anabolic effects on muscle protein metabolism are beneficial in chronic liver disease. GH-deficient rats showed impaired mitochondrial morphology, with IGF-I reversing these abnormalities [30]. IGF-I also mitigates oxidative stress and improves mitochondrial function, as IGF-I administration reduces oxidative mitochondrial damage, corrects mitochondrial function impairments, and decreases caspase activities [32]. Consistent with these findings, IGF-I administration improved liver dysfunction and fibrosis in a rat cirrhosis model and mitochondrial function in aging rats [33].

In humans, MASLD correlates with low circulating IGF-I levels [34,35], with IL-6 and IGF-I serving as independent prognostic factors for liver steatosis and MASH in morbidly obese patients [36]. Hyaluronic acid levels, a fibrotic marker, negatively correlate with IGF-I and the IGF-I/IGFBP-3 ratio in patients with MASLD [37]. While GH has a direct role in hepatocytes regarding anti-steatosis and gene expression [37,38,39], these findings suggest that IGF-I exerts GH-independent effects in the liver through various mechanisms [12,13]. Collectively, GH and IGF-I are crucial in liver function, influencing hepatocytes, macrophages, and hepatic stellate cells to counteract steatosis and fibrosis progression (Figure 3).

### 3.4. SGA Impact on MASLD Development

The patient was born at full term but had a low birth weight of 2320 g and was an SGA infant. Ibanez et al. [40] found that SGA children between the ages of two and six years gained more total and abdominal fat and had greater increases in insulin, IGF-I, and neutrophil-to-lymphocyte ratio, compared to appropriate-for-gestational-age (AGA) children. At six years old, the average amount of visceral fat in SGA children was >50% higher than that in AGA children. 

Soto et al. [41] found that the fasting insulin concentration in one-year-old babies was significantly higher in SGA infants with catch-up growth than in those without catch-up growth and AGA infants. These data indicate that pathophysiological mechanisms linking prenatal growth and postnatal sensitivity to insulin are present at as early as one year old [42].

A case report published in 1997 noted improvements in fatty liver associated with panhypopituitarism after GH administration in full-term but low-birth-weight infants, suggesting that fatty liver is at least partly attributable to GH deficiency [43]. Persons with low birth weights or who were thin at birth have a high prevalence of insulin resistance or metabolic syndrome, with the co-existence of glucose intolerance, hypertension, and hypertriglyceridemia. Several recent studies suggested that insulin resistance could lead to other metabolic disorders in children, adolescents, and adults born as SGA infants, including type 2 diabetes, dyslipidemia, and MASLD. Therefore, SGA birth is thought to be a risk factor for MASLD [44,45,46,47,48,49]. Ultimately, it is thought that progression to MASLD/MASH may begin in childhood for children born as SGA infants if they have GH deficiency.

### 3.5. Adult GH Deficiency (AGHD)

In this case, the patient required continued GH injections into adulthood, but GH therapy under health insurance for AGHD was only approved in Japan in 2009 (when the patient was 42 years old). In 2004, clinical trials in Japanese patients with AGHD confirmed that GH administration improves body composition and serum cholesterol profiles [50].

## 4. Conclusions

Accumulating evidence clearly demonstrates that MASLD/MASH presents critical complications of both adult and childhood GH deficiency, which can worsen an individual’s prognosis. This case emphasizes the importance of continuous GH supplementation from childhood to adulthood in a patient with childhood-onset GH deficiency.

## Data Availability

Data is contained within the article.
